# Dynamics of opinion formation under majority rules on complex social networks

**DOI:** 10.1038/s41598-019-57086-3

**Published:** 2020-01-16

**Authors:** Vu Xuan Nguyen, Gaoxi Xiao, Xin-Jian Xu, Qingchu Wu, Cheng-Yi Xia

**Affiliations:** 1 School of Electrical and Electronic Engineering, Nanyang Technological University, Singapore, 639798 Singapore; 2 Complexity Institute, Nanyang Technological University, 18 Nanyang Drive, Singapore, 637723 Singapore; 3 Department of Mathematics, Shanghai University, Shanghai, 200444 China; 4 College of Mathematics and Information Science, Jiangxi Normal University, Jiangxi, 330022 China; 5 School of Computer Science and Engineering, Tianjin University of Technology, Tianjin, 300384 China

**Keywords:** Computational science, Statistical physics, thermodynamics and nonlinear dynamics

## Abstract

We study opinion dynamics on complex social networks where each individual holding a binary opinion on a certain subject may change her/his mind to match the opinion of the majority. Two rules of interactions between individuals, termed as *classic majority* and *influence majority* rules, respectively, are imposed on the social networks. The former rule allows each individual to adopt an opinion following a simple majority of her/his immediate neighbors, while the latter one lets each individual calculate the influence of each opinion and choose to follow the more influential one. In this calculation, the influences of different opinions are counted as the sum of the influences of their respective opinion holders in neighborhood area, where the influence of each individual is conveniently estimated as the number of social connections s/he has. Our study reveals that in densely-connected social networks, all individuals tend to converge to having a single global consensus. In sparsely-connected networks, however, the systems may exhibit rich properties where coexistence of different opinions, and more interestingly, multiple steady states of coexistence can be observed. Further studies reveal that low-degree and high-degree nodes may play different roles in formulating the final steady state, including multi-steady states, of the systems under different opinion evolution rules. Such observations would help understand the complex dynamics of opinion evolution and coexistence in social systems.

## Introduction

Social opinion evolution and opinion formation have been extensively studied in the past decades^1–8^. A popular approach adopted in such studies is to model social opinions into a binary system^2,3,9–11^. That is, we assign two different values, typically denoted as 0 and 1, respectively, to reflect two different opinions. Such a model could conveniently resemble those cases where there exist two competing opinions, e.g., approval and disapproval in a vote or selection between two candidates in an election. In such a system, local interactions between individuals may drive the system to an equilibrium in the final steady state where a certain opinion dominates the system or coexists with the other one. In social opinion formation, people may be affected by those who are socially connected to them (e.g., friends, family members, and colleagues *et al*.) and tend to adopt the opinion that the majority of their connected individuals hold. To reflect such “majority effects”, a well-known *majority dynamics model* (also known as *local majority rule* or *majority voter model*) has been proposed^10–14^. Under the rule, each individual holds an initial opinion. During the temporal evolution, the individuals iteratively update their states to match the opinion of the majority of her/his immediate neighbors. In this paper, we term this rule as the *classic majority rule*, to differentiate it from the new majority rule we shall introduce later in this paper.

The classic majority rule, simple as it is, exhibits rich dynamical properties which have inspired scientists to propose and investigate quite a few variant models. The works by Mossel and Tamuz *et al*.^10,12^ focus on effective aggregation of information on two-state systems under majority dynamics and the conditions that drive the social systems to converge to unanimity. It also addresses other questions such as whether an opinion supported by the initial majority remains as the majority in the final state. Studies have also been carried out to develop different theoretical frameworks with different configurations of underlying networks. Some examples include the studies by Kanoria and Montanary on majority dynamics on regular tree graphs^9^, by Howard on 3-regular trees^15^, and by Fontes, Schonmann, and Sidoravicius^16^ on *Z*^*d*^, etc.

In most studies, complete consensus state at which all individuals adopt the same opinion is observed at the end of the opinion evolution process. The study in^11^ shows that under the majority dynamics, when the initial opinions of agents on Erdös-Rényi random graphs with degrees $\Omega (\sqrt{n})$ are independently and identically distributed, the agents eventually converge to the initial majority opinion, with a constant probability. It is also argued in^17^ that independently of underlying graphs, there is always a group formed by a half of the agents that can obliterate the opposite opinion. Likewise, the authors in^12^ show that if the initial population is sufficiently biased towards a certain opinion, this opinion may finally become the unanimous preference of the entire population. Some other variants of majority dynamics can be found in^13,18–21^.

In our study, for the first time to the best of our knowledge, we find that under the classic majority rule, the systems’ behaviors may be different in densely and sparsely connected networks, respectively. Specifically, the existence of a large proportion of low-degree nodes may sustain the coexistence of two competing opinions in equilibrium, which is not observed in dense networks. These low-degree nodes are connected to each other forming up separating boundaries between clusters of opposite opinion holders. As the setting up of such separating boundaries is sensitive to the initial allocations of different opinion holders, multi-steady states of opinion coexistence can be observed.

We then further consider the fact that when people choose to follow the “majority” opinion, what matters most may not be the number of people holding each of the opinions, but how influential each opinion is. In real life, the influence of an opinion may be estimated, say, by roughly calculating the sum of the influences of those opinion holders supporting this opinion. While there could be many different ways for people to estimate the influence of each opinion holder, a visible metric people may intentionally or unintentionally use in their calculations is the number of “valid” social connections this individual has, through which this individual may influence other people. In this report, we propose a new majority game model, termed as *influence majority rule*. Specifically, we let each individual follow the opinion with a stronger overall influence among his/her immediate neighbors. As a simplest case, we let the overall influence of an opinion be equal to the sum of the influences that its holders have, where the influence of each neighbor equals the number of connections s/he has. In a complex social network model where each individual is represented as a vertex and the connections s/he has are represented as network edges, this means that for each node, we sum up the nodal degrees of its immediate neighbors holding opinions 0 and 1 respectively; and let this node adopt the opinion with a larger sum value. Other influence majority rules certainly can also be proposed, e.g., by assigning a higher-degree node an influence value that is nonlinearly proportional to its degree, or by measuring or estimating every node’s betweenness, etc. Studies on these models, however, are out of the scope of this paper and shall be carried out in future research work.

Under the influence majority rule, as will be presented later, at first sight, it may seem that the observations are similar to those under the classic majority rule. That is, in dense networks, complete consensus would be achieved at the end of opinion evolution, while in sparse networks, multi-steady states of coexistence would be achieved. Closer observations, however, reveal that the seemingly similar final states have different structures and they emerge due to different reasons: unlike that under the classic majority rule where low-degree nodes form up separating boundaries between clusters holding opposite opinions, under the influence majority rule, it is high-degree hub nodes that connect their surrounding low-degree nodes to form into communities holding different opinions.

The rest of the paper is organized as follows. First, we present a formal description of the system model and the opinion formation rules, followed by some brief discussions on theoretical analyses. Then simulation results and discussions are presented. Finally we conclude the paper and briefly discuss some possible future research.

## Models and Analyses

### System models and opinion formation rules

Let *G* = (*V*, *E*) be an undirected graph representing a social network, where *V* is the set of vertices (nodes) representing individuals and *E* is the set of edges (links) representing social connections. Denote the set of neighbors of a vertex *v* ∈ *V* as *η*(*v*) and the nodal degree of *v* as *k*_*v*_, *k*_*v*_ = |*η*(*v*)|. At time step *t*, each node *v* ∈ *V* holds a state *S*_*v*_(*t*) ∈ {0, 1} representing one’s opinion on a particular subject.

At the start of the opinion dynamics, each node *v* is endowed with a state *S*_*v*_(0) drawn from {0, 1} at a certain distribution as assigned. At each time step, a node is randomly chosen and its state is updated following either of the two majority rules. Iteratively, the nodes update their states over time until an equilibrium is achieved where no vertex needs to further change its state.

Under the classic majority rule, a vertex shall adopt the opinion that occupies the majority of its adjacent nodes. When there is a tie, i.e., when the numbers of adjacent nodes holding each of the two opinions equal each other, the node shall retain its current opinion. When under the influence majority rule, a vertex shall sum up the degrees of those adjacent nodes holding opinions 0 and 1, respectively. It shall adopt the opinion that has a larger value of the sum. When there is a tie, the node shall retain its current opinion.

Mathematically, the two different rules can be respectively described in Eqs. (1) and (2) as follows:

1 $${S}_{v}(t)={argma}{{x}}_{s\in \{0,1\}}|\{u|{S}_{u}(t-1)=s,u\in \eta (v)\}|,$$

2 $${S}_{v}(t)={argma}{{x}}_{s\in \{0,1\}}\,\sum _{w\in \{u|{S}_{u}(t-1)=s,u\in \eta (v)\}}{k}_{w}.$$

For the sake of convenience, we hereafter term the classic majority rule and the influence majority rule as CMR and IMR, respectively.

### System dynamics equations

Denote the relative densities of *k*-degree nodes holding states 0 and 1 at time t as *ρ*_0,*k*_(*t*) and *ρ*_1,*k*_(*t*) = 1 − *ρ*_0,*k*_(*t*), respectively. In an uncorrelated random network with an arbitrary degree distribution *P*(*k*), the temporal evolution of the system governed by CMR can be approximately described by the following dynamics equations:

3 $$\frac{d{\rho }_{0,k}(t)}{dt}=-\,{\rho }_{0,k}(t)\,\mathop{\sum }\limits_{m=0}^{\lfloor \frac{k-1}{2}\rfloor }\,(\begin{array}{l}k\\ m\end{array}){\theta }_{0}{(t)}^{m}{\theta }_{1}{(t)}^{k-m}+{\rho }_{1,k}(t)\,\mathop{\sum }\limits_{m=0}^{\lfloor \frac{k-1}{2}\rfloor }\,(\begin{array}{l}k\\ m\end{array}){\theta }_{1}{(t)}^{m}{\theta }_{0}{(t)}^{k-m},$$

where *θ*_0_(*t*) (*θ*_1_(*t*)) is the probability that a randomly chosen link points to a 0-state node (1-state node):

4 $$\begin{array}{rcl}{\theta }_{0}(t) & = & \frac{{\sum }_{k}\,kP(k){\rho }_{0,k}(t)}{{\sum }_{k}\,kP(k)}\\  & = & \frac{{\sum }_{k}\,kP(k){\rho }_{0,k}(t)}{\langle k\rangle },\end{array}$$

where 〈*k*〉 is the average degree,

5 $${\theta }_{1}(t)=1-{\theta }_{0}(t).$$

To derive the dynamics equation for IMR, let *D*_*l*_ be a multiset having cardinality *l* drawn from an ordinary set $U={\mathbb{N}}\cap [{k}_{{\rm{\min }}},{k}_{{\rm{\max }}}]$. *D*_*l*_ can be represented by a set of ordered pairs: ${D}_{l}=\{(k,{n}_{{D}_{l}}(k))|k\in U,{n}_{{D}_{l}}(k)\in {\mathbb{N}}\}$, where ${n}_{{D}_{l}}(k)$ denotes the number of occurrences of the element *k* in *D*_*l*_ and hence ${\sum }_{k\in {\rm{Supp}}({D}_{l})}{n}_{{D}_{l}}(k)=l$. Here ${\rm{Supp}}\,({D}_{l})=\{k\in U|{n}_{{D}_{l}}(k) > 0\}$ is the support set of *D*_*l*_ formed from distinct elements of *D*_*l*_. Denote the total sum of elements of *D*_*l*_ as ${\sum }_{{D}_{l}}={\sum }_{k\in {\rm{Supp}}({D}_{l})}k{n}_{{D}_{l}}(k)$ and define function $\Gamma (.\,)$ of *D*_*l*_ as $\Gamma ({D}_{l})={\prod }_{k\in {\rm{Supp}}({D}_{l})}{n}_{{D}_{l}}(k)!$. The dynamics equation of a system governed by IMR can be expressed as follows:

6 $$\begin{array}{ccc}\frac{d{\rho }_{0,k}(t)}{dt} & = & -{\rho }_{0,k}(t)k!\mathop{\sum }\limits_{m=0\,}^{k-1}\sum _{\Sigma {D}_{m} < \Sigma {D}_{k-m}}\frac{1}{\Gamma ({D}_{m})\Gamma ({D}_{k-m})}\prod _{{k}_{n}\in {\rm{S}}{\rm{u}}{\rm{p}}{\rm{p}}({D}_{m})}\,{{\theta }_{0,{k}_{n}}}^{{n}_{{D}_{m}}({k}_{n})}(t)\\  &  & \times \,\prod _{{k}_{n}\in {\rm{S}}{\rm{u}}{\rm{p}}{\rm{p}}({D}_{k-m})}\,{{\theta }_{1,{k}_{n}}}^{{n}_{{D}_{k-m}}({k}_{n})}(t)+{\rho }_{1,k}(t)k!\mathop{\sum }\limits_{m=0\,}^{k-1}\sum _{\Sigma {D}_{m} < \Sigma {D}_{k-m}}\frac{1}{\Gamma ({D}_{m})\Gamma ({D}_{k-m})}\\  &  & \times \,\prod _{{k}_{n}\in {\rm{S}}{\rm{u}}{\rm{p}}{\rm{p}}({D}_{m})}\,{{\theta }_{0,{k}_{n}}}^{{n}_{{D}_{m}}({k}_{n})}(t)\prod _{{k}_{n}\in {\rm{S}}{\rm{u}}{\rm{p}}{\rm{p}}({D}_{k-m})}\,{{\theta }_{0,{k}_{n}}}^{{n}_{{D}_{k-m}}({k}_{n})}(t),\end{array}$$

where ${\theta }_{0,{k}_{n}}(t)$ (${\theta }_{1,{k}_{n}}(t)$) is the probability that a randomly chosen link points to a node having degree *k*_n_ and state 0 (1) at time *t*:

7 $${\theta }_{0,{k}_{n}}(t)=\frac{{k}_{n}P({k}_{n}){\rho }_{0,{k}_{n}}(t)}{\langle k\rangle },$$

8 $${\theta }_{1,{k}_{n}}(t)=\frac{{k}_{n}P({k}_{n})}{\langle k\rangle }-{\theta }_{0,{k}_{n}}(t).$$

Note that when the network has a delta-Dirac degree distribution, Eq. (6) is reduced to Eq. (3).

## Simulation Results and Discussions

We investigate the evolution of the system state under the two majority rules, mainly on underlying scale-free networks^22^ generated from the configuration method^23^. Specifically, let *F*_0_(*t*) and *F*_1_(*t*) denote the fractions of nodes holding states 0 and 1 at time step *t*, respectively. At *t* = 0, *F*_0_(0) ≤ 0.5 of the population are randomly chosen to be endowed with state 0 and the remaining nodes are endowed with state 1. The system starts to evolve under either of the two rules until the final steady state is reached. We refer to the state with a larger (smaller) proportion in a specified graph as the majority (minority) state of that graph.

**Figure 1:**
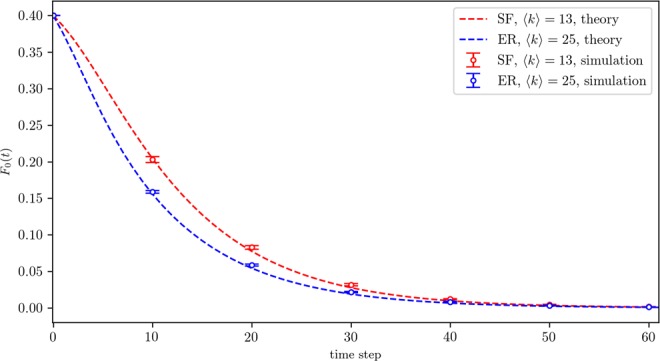
Temporal fraction of 0-state nodes under classic majority rule on scale-free (SF) and Erdös-Rényi (ER) networks with a size of *N* = 20000, *k*_*min*_ = 3, and *k*_*cutoff*_ = 70. The simulation results are averaged over 50 independent realizations with error bars representing the standard deviation.

We first verify Eq. (3) by carrying simulations on scale-free and Erdös-Rényi (ER)^24^ networks. Figure 1 shows the decline of the initial minority state over time, finally leading to the unanimity among all individuals in the steady state. Reasonably good matches between analytical and simulation results can be observed. Extensive simulations also show that the denser the networks are (quantified by average degree $\langle k\rangle$), the faster the state converges. This observation is in line with the conclusions in^13^. It implies that having more social connections may facilitate exposing a node to the community holding the majority opinion and promote the updating of its state to match the dominant state of its neighbors, resulting in a faster concurrence on a denser network.

**Figure 2:**
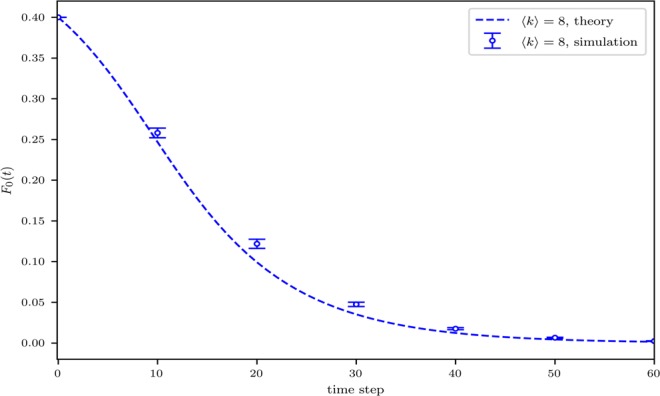
Temporal fraction of 0-state nodes under the influence majority rule on ER networks with a size of *N* = 20000, *k*_*min*_ = 5, and *k*_*cutoff*_ = 10. The simulation results are averaged over 50 independent realizations with error bars representing the standard deviation.

Under IMR, for a general network with a wide range of nodal degrees, the space of multisets becomes massive and consequently, the calculations of Eq. (6) may require heavy computations. For simplicity, we present both simulation and analytical results on ER networks with a relatively narrow nodal degree range where *k* varies from 5 to 10. As shown in Fig. 2, there exists a reasonably good match between theoretical analysis and numerical simulation results, and the vanishment of the initial minority is observed.

**Figure 3:**
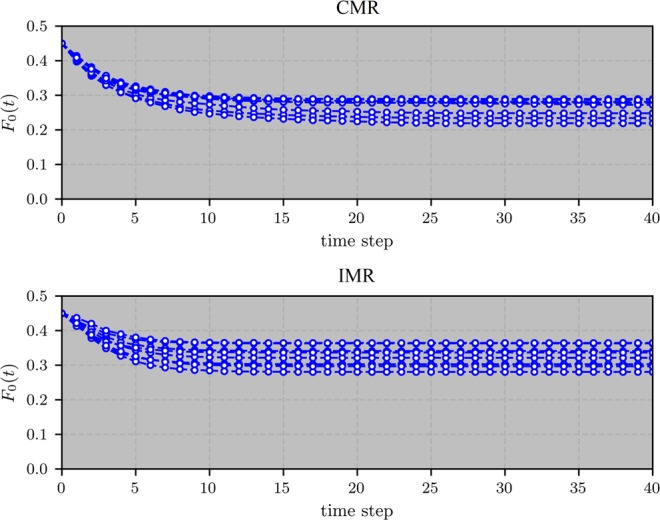
The fractions of nodes with state 0 in time on a scale-free network with a size of *N* = 20000 and an average degree of $\langle k\rangle =4.5$ under CMR and IMR, respectively. Each curve corresponds to simulation results on the same network with the same parameters but a different randomly-generated initial set of 0-state nodes.

More noticeably, the observation that the initial majority opinion tends to completely dominate the whole network in the final state does not hold in sufficiently sparse networks. As illustrated in Fig. 3, the state of minority may persist to exist in the steady state where the coexistence of the two states maintains stable.

The study in^13^ shows that for 4-regular infinite graphs with the minimum nodal degree *k*_*min*_ ≥ 5, the network state under the classic majority rule shall converge to unanimity. We observe that when *k*_*min*_ < 5 and the networks are sufficiently sparse, the coexistence of two states may sustain under both rules. As to why there could be a coexistence of different states, we argue that the main reasons are different for different rules. For CMR, it is because that the interconnected low-degree nodes could form up separating boundaries between communities favoring opposite opinions.

Figure 3 shows that the fraction of the minority state decreases over time and finally remains unchanged where the state coexistence persists. This occurs only on sparse networks where low-degree nodes constitute a reasonably large portion of the population. The low-degree nodes holding minority state may thus spread widely across the network with a good chance to be connected to each other, forming up inter-community separating boundaries between communities holding the opposite opinions. In a densely connected network, on the other hand, the network nodes are with higher degrees, giving them a better chance to be connected to many nodes of different states and consequently, quickly adopt the state of majority. A separating boundary formed up by low-degree nodes holding a minority state can hardly sustain under such case.

**Figure 4:**
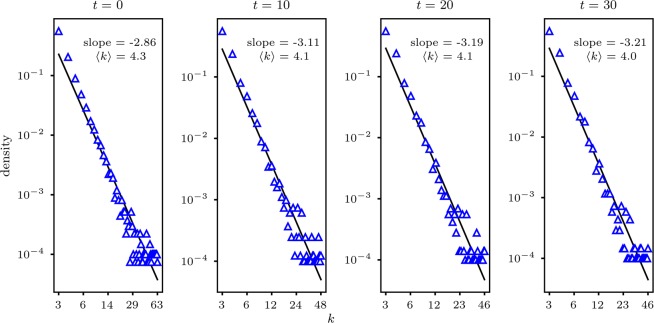
Temporal distribution of degrees of minority-state nodes in log-log scale from the initial state to the final steady state. A scale-free network of a size of *N* = 20000 and an average degree of $\langle k\rangle =4.5$ is employed. The fraction of the minority in the initial and final state is 0.45 and 0.2, respectively. The best-fitting lines are obtained by using linear least-squares regression.

**Figure 5:**
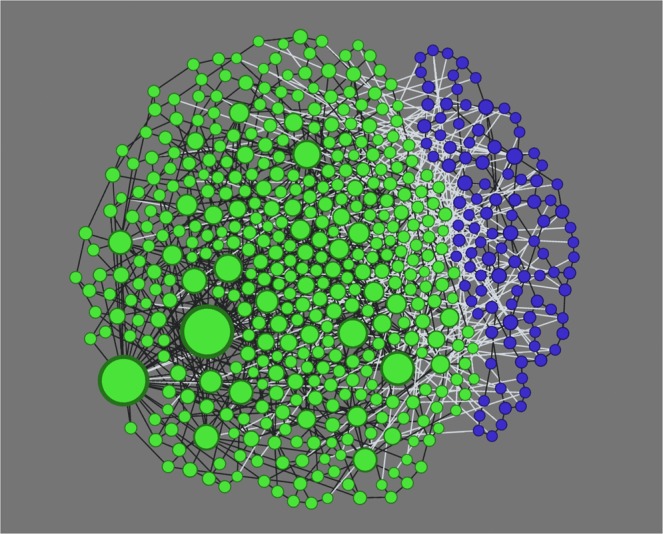
A snapshot of a 500-node network in the steady state governed by CMR. Peripheral nodes of the minority (blue) are mostly of low degrees and connected with each other (via black links), forming up a sustained minority group while being exposed to the majority (green) (via white links). Size of each node reflects its nodal degree.

We illustrate in Fig. 4 an example showing how low-degree nodes take an increasingly larger proportion in a minority group while the overall group size decreases over time until a stable structure is reached. The decline of the average degree, as well as the steeper slope of the best-fitting line, reflects an increasing proportion of low-degree nodes in the minority community during the dynamic process. This process comes to an end when the population reaches an equilibrium where the two states coexist. Figure 5 illustrates such a coexistence of opposite states in a sparse network. Due to the limited size of the network, only a single community of majority-opinion holders is observed. Nevertheless, we could see that minority-opinion holders are connected to each other and form up their own sustained community.

**Figure 6:**
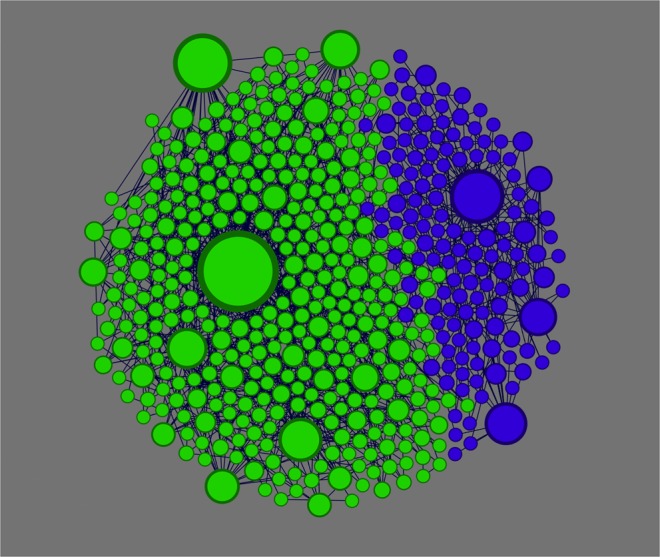
A snapshot of a 500-node network in the steady state governed by IMR. The two state communities are formed up, each containing its own hubs that sustain the persistence of the two communities. Size of each node reflects its nodal degree.

Under the regime of IMR, as illustrated in Fig. 2, global consensus would be achieved in dense networks. In sparse networks, as can be observed in Fig. 3 (IMR), majority and minority states coexist. Note that, though the two subfigures of Fig. 3 may appear to be similar to each other at the first sight, the coexistences of opposite opinions under the two different majority rules are due to different reasons. Specifically, under IMR, highly connected hub nodes play a critical role. The communities of minority-opinion holders center around some hub nodes while most of the other nodes of the communities are directly linked to those hubs (see Fig. 6). These hubs keep the state of their neighbors difficult to be changed according to the rule: In the community of nodes holding the minority state, a low-degree node has a small number of links. At least one of these links connects to one of the hubs of the minority state while most of the other links possibly connect to nodes in majority communities but not their hubs. Under such case, nodes holding the minority state may persist to exist in sparse networks. In dense networks, on the other hand, a minority-state node, even when it is connected to a hub of its own state, may still have a good chance to connect to one or more high-degree nodes of the majority-state communities, giving it a good chance to change its state. This may lead to a series of events where nodes leave the minority communities, ultimately causing the vanishment of the minority state.

**Figure 7:**
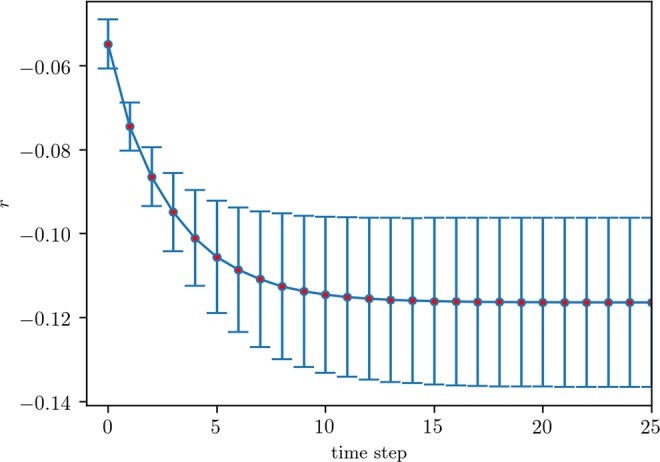
Temporal assortativity coefficient of 0-state community on a network with a size of *N* = 30000, *k*_*min*_ = 3, *k*_*cutoff*_ = 70 and *k* = 5. *F*_0_(0) = 0.45. The simulation results are averaged over 50 independent realizations, 10 on each of 5 randomly generated networks, with error bars representing the standard deviation.

From the above discussions, we may also expect that, in sparse networks under IMR, a minority-state holder not linked to a minority-state hub may tend to change its state. The communities of the two states shall thus become more and more disassortative until they get stabilized. We verify such is indeed the case by measuring the assortativity of the minority-state communities. Specifically, we remove nodes holding the majority state and then compute the assortativity coefficient of the remaining subgraph using the following equation^25^:

9 $$r=\frac{{\sum }_{xy}\,xy({e}_{xy}-{a}_{x}{b}_{y})}{{\sigma }_{a}{\sigma }_{b}},$$

where *e*_*xy*_ is the fraction of edges in the graph that connect a node of degree *x* to another one of degree *y*, *a*_*x*_ = ∑_*y*_ *e*_*xy*_, *b*_*y*_ = ∑_*x*_ *e*_*xy*_, *σ*_*a*_ and *σ*_*b*_ are the standard deviations of the distributions *a*_*x*_ and *b*_*y*_, respectively. Figure 7 shows an example where the assortativity coefficient decreases as the opinion evolution goes on.

**Figure 8:**
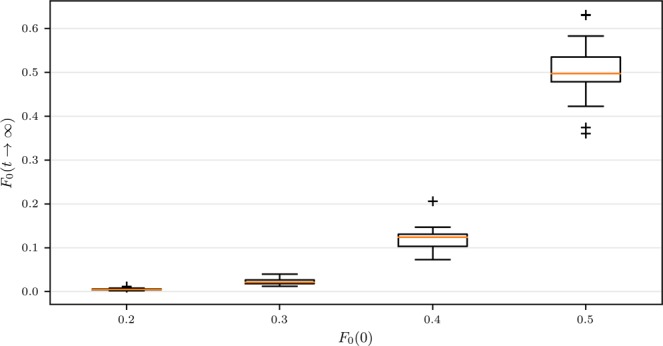
Box plots showing the statistical fraction of 0-state nodes in the initial state versus that in the steady state. Simulations are carried out on networks with a size of *N* = 100000, *k*_*min*_ = 3, *k*_*cutoff*_ = 70 and an average degree of *k* = 4.5. The results for each *F*_0_(0) are obtained from 50 independent realizations: we generate 5 random scale-free networks and carry out 10 realizations of simulations on each of them, each realization has an independent random allocation of initial state on network nodes.

Figure 8 further shows the steady-state fraction of nodes with state 0 in respect of its portion at the beginning. It reveals that the larger proportion the minority opinion initially takes, the wider range the coexistence may finally have. More noticeably, if the initial minority and majority are sufficiently close to each other (including the special case where each of them occupies 50% of the population at the beginning), the initial minority state may indeed have a chance to become the majority state in the end. Our numerical simulation experiences show that this is most likely to happen when the largest hubs hold the minority state at the beginning of the evolution. Note that the relatively large standard deviations in Fig. 7 and the relatively wide ranges of the box plots in Fig. 8 mainly come from the existence of multi-steady states, as will be discussed below. Also note that in the ER networks, since there are not obvious hub nodes with very high degrees, the evolution dynamics under IMR appears to be similar to, though not exactly the same as, that under CMR.

Lastly, it is worth pointing out that Fig. 3 reveals an important phenomenon that can be observed in sparse networks governed by either of the two majority rules. That is, both rules may drive the systems to *multiple* steady states. Specifically, with the same initial percentage of the minority state with independent random initial state allocation, on the same underlying network, the evolution may end up with different final states capturing different portions of the population. This is mainly due to the randomness of the decline of the minority under the majority effects. To be specific, “majority-oriented” individuals facilitates the dominant growth of the initial majority opinion in dense networks, generally resulting in the vanishment of the minority. In a sparse network, groups formed by interconnected minority-opinion holders are more likely to form up and sustain. Such minority-opinion groups, however, could be vulnerable to small fluctuations of the system state, allowing them to be “swallowed” by the majority-opinion communities. For example, under CMR, when a small number of minority-opinion holders change their mind, say, when slightly more than half of their neighbors are majority-opinion holders, cascading effects may cause more minority-opinion holders to change their mind, leading to a significant decline or even total vanishment of a minority community. The vulnerability hence results in the multi-steady states at the end of the system evolution.

Such vulnerability of minority-opinion communities also exists under IMR, but to a less extent, as it typically takes changing the opinion of a hub to eliminate a minority-opinion community under IMR. This may explain why in sparse networks, minority state tends to occupy a relatively larger portion of nodes at final steady state under IMR than that under CMR, as can be observed in Fig. 3.

## Conclusion

In this report, motivated by the observations that though people may tend to follow majority in opinion formation, they may have different ways in evaluating which opinion is the majority one, we studied the opinion formation under the classic majority rule and the influence majority rule, respectively. It is found that under both rules, in dense networks, global consensus could be steadily achieved, while in sparse networks, multi-steady states of opinion coexistence may be observed. Closer observations, however, showed that the coexistences of opposite opinions under the two rules are caused by different reasons: interconnected low-degree minority-state nodes form into their own clusters under the classic majority rule, while hub nodes may play a critical role in forming up different communities under the influence majority rule. Our studies reveal some useful insights into how different opinions manage to coexist and sustain in complex social systems.

When social systems are arguably becoming more and more densely connected, theoretically speaking it may become easier than before to achieve global consensus, while co-existence of different opinions will almost for sure still be observed everywhere in real life. Such coexistences may be more and more caused by other factors, e.g., the existence of community structures, the temporal evolution of the systems, the adoption of different influence evaluation methods by different individuals, and some individuals/groups’ strong resistances to the majority effects, etc. Studies on the impacts of such effects in synthetic and real-life networks shall be of our future research interest.
